# A Comparative Study in Real-Time Scene Sonification for Visually Impaired People

**DOI:** 10.3390/s20113222

**Published:** 2020-06-05

**Authors:** Weijian Hu, Kaiwei Wang, Kailun Yang, Ruiqi Cheng, Yaozu Ye, Lei Sun, Zhijie Xu

**Affiliations:** 1 National Engineering Research Center of Optical Instrumentation, Zhejiang University, Hangzhou 310058, China; huweijian@zju.edu.cn (W.H.); rickycheng@zju.edu.cn (R.C.); yaozuye@zju.edu.cn (Y.Y.); leo_sun@zju.edu.cn (L.S.); 2 Institute for Anthropomatics and Robotics, Karlsruhe Institute of Technology, 76131 Karlsruhe, Germany; kailun.yang@kit.edu; 3 School of Computing and Engineering, University of Huddersfield, Huddersfield HD1 3DH, UK; z.xu@hud.ac.uk

**Keywords:** electronic travel aid, visually impaired people, sonification, scene detection

## Abstract

In recent years, with the development of depth cameras and scene detection algorithms, a wide variety of electronic travel aids for visually impaired people have been proposed. However, it is still challenging to convey scene information to visually impaired people efficiently. In this paper, we propose three different auditory-based interaction methods, i.e., depth image sonification, obstacle sonification as well as path sonification, which convey raw depth images, obstacle information and path information respectively to visually impaired people. Three sonification methods are compared comprehensively through a field experiment attended by twelve visually impaired participants. The results show that the sonification of high-level scene information, such as the direction of pathway, is easier to learn and adapt, and is more suitable for point-to-point navigation. In contrast, through the sonification of low-level scene information, such as raw depth images, visually impaired people can understand the surrounding environment more comprehensively. Furthermore, there is no interaction method that is best suited for all participants in the experiment, and visually impaired individuals need a period of time to find the most suitable interaction method. Our findings highlight the features and the differences of three scene detection algorithms and the corresponding sonification methods. The results provide insights into the design of electronic travel aids, and the conclusions can also be applied in other fields, such as the sound feedback of virtual reality applications.

## 1. Introduction

According to the World Health Organization, around 253 million people live with vision impairments in the world [1]. Visually Impaired People (VIP) meet various difficulties when they travel in unfamiliar environment due to their visual impairments. In the late twentieth century, with the development of semiconductor sensors and portable computers, a broad range of Electronic Travel Aids (ETAs) were proposed to help VIP perceive environments and avoid obstacles [1].

Early ETAs usually use ultrasonic sensors to detect obstacles and remind VIP through vibration or beeps [2,3]. Due to the low spatial resolution, ultrasonic sensors can only acquire limited information in every single measurement, which is insufficient for VIP to perceive environments in real time. In the past few years, we have witnessed the rapid development of RGB -Depth (RGB-D) cameras and the wide applications in consumer electronics, robotics as well as other fields, because of their versatility, compact volume, low power consumption and low cost [4,5,6]. Furthermore, efficient computer vision algorithms and the increase of computation power also provide attractive possibilities to achieve real-time scene detection on mobile platforms [7,8]. However, there is still no major breakthrough in the field of human-computer interaction, which limits the overall performance of ETAs.

Hearing and touch are two main senses that can be utilized to realize the interaction between VIP and ETAs. Intuitively, auditory-based interaction methods have more potential considering the capability of conveying complex scene information, since sound has more dimensions that can be utilized, such as pitch, loudness and timbre. On the other hand, auditory-based interaction methods should be appropriately designed because hearing is the primary way for VIP to perceive surrounding environments. A practical interaction method is expected to convey sufficient information without imposing extra burdens to VIP. Towards this end, we introduce sonification to our studies. Sonification involves a series of researches of rendering sounds in response to data [9]. The theories and techniques of sonification can guide us to design practical and effective interaction methods.

**Figure 1:**
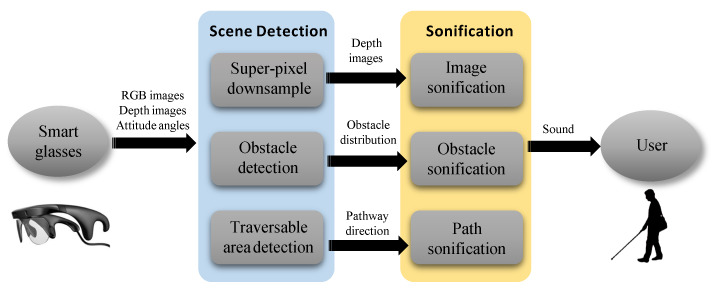
Flow chart of the proposed assistance system. We propose three sonification methods and corresponding scene detection algorithms to convert scene information to sound.

In this paper, we present three independent auditory-based interaction methods to convey scene information to VIP, as shown in Figure 1. We use a pair of smart glasses with an RGB-D camera and an attitude sensor as our hardware platform to acquire environmental information for all three methods. The first sonification method aims to convey raw depth images to VIP. We utilize the super-pixel segmentation algorithm [10] to reduce the size of depth images and make use of a simple but useful sonification method to convert depth information to sound. The second method detects obstacles in the field of view and conveys the distribution of obstacles to VIP. The third method is based on the traversable area detection algorithm [11] and conveys the direction of pathway to VIP. In addition, a comprehensive user study including a field experiment and a questionnaire survey was also performed to compare the usability of the three sonification methods. The main contribution of this paper is that we compare the effects of different levels of information in assisting VIP, which provides insights into the design of electronic travel aids.

This rest of this paper is organized as follows. Section 2 reviews some interaction methodologies used in ETAs. Section 3 describes our sonification methods in detail. Section 4 and Section 5 present the user study and the results. Section 6 draws the conclusions.

## 2. Related Work

In the literature, the interaction method of ETAs can be divided into two categories: haptic-based interaction and auditory-based interaction.

Haptic-based interaction methods are usually implemented by micro vibration motors or electrodes, and with the help of ultrasonic range sensors, many low-cost ETAs have been proposed [12]. In 2015, B. Ando et al. proposed an ETA based on a cane [13]. They used ultrasonic range sensors to detect obstacles and deployed six eccentric mass motors to convey range information. Another research put three acoustic range sensors on a belt and used a bracelet with two vibration motors to alert VIP [14]. Although ultrasonic sensors have the characteristics of low cost and low power, they alone are not enough to provide comprehensive assistance to VIP. In contrast, camera can capture much more information and have been widely used in ETAs. In 2011, Y. H. Lee and G. Medioni proposed a camera-based ETA to assist VIP to navigate in indoor environments [15]. They integrated four vibration motors in a vest and used the vibration combinations of four motors to convey navigation information, such as turn left, turn right and go ahead. Some haptic-based interaction methods are also complex. P. Bach-y-Rita et al. designed a 49-point electrotactile stimulus array and placed it on the tongue to interact with the user [16]. Some basic tactile patterns were tested on five participants to study the interaction performance. BrainPort V100 [17] is a commercial ETA based on this idea. It contains a camera mounted on a pair of sunglasses and a tongue array with 400 electrodes, and the whiter the pixel value in the image captured by the glasses, the stronger the stimulus yielded by the corresponding electrode.

Compared with haptic-based interaction, auditory-based interaction is different in many aspects. Firstly, hearing is the only way for VIP to perceive remote surroundings. They usually estimate the distance and direction of traffic by hearing to avoid injuries when crossing the street. Thus, auditory-based interaction methods need to be carefully designed to convey necessary information and not interfere with VIP’s hearing from surroundings as much as possible. Secondly, there are nearly thirty auditory dimensions available for sonification [18]. This means there are thousands of combinations of auditory dimensions can be deployed to encode real-world scenes. The choice of auditory dimensions has a great influence on the feasibility of sonification methods, which makes auditory-based interaction more flexible and challenging. Based on the above discussions, this paper focuses on auditory-based interaction.

Some articles focused on scene detection algorithms and proposed some wearable prototypes along with an auditory-based interaction method. A. Rodríguez et al. proposed an obstacle detection and warning system using acoustic feedback [19]. They designed an RGB-D camera-based ground plane estimation algorithm to segment the ground from images and extract potential obstacles. The detected obstacles were represented in a polar grid and a sonification method was designed to convert the polar grid into beeps. The beep frequency and the number of repetitions were utilized to represent the position of obstacles in the polar grid. In 2014, A. Aladrén et al. described a robust algorithm that detects and classifies the main structural elements of current scene based on an RGB-D camera [20]. Following the perception pipeline, an auditory-based interaction method was also provided. They used speech instructions to describe the surrounding environment and utilized stereo beeps with different frequencies to represent the distance to obstacles. The main drawback of these articles is that they mainly focus on obstacle detection algorithms and do not analyze the impact of interaction methods on system usability.

Another important cluster of articles focused more on auditory-based interaction methods and conducted many experiments to illustrate the effectiveness of the interaction methods. VOICE is one of the most famous image sonification method [21]. It converted grayscale images to sound by scanning it from left to right while associates elevation with pitch and brightness with loudness. VOICE did not rely on complex image processing algorithms and it can be ported to many platforms, such as smart phones and wearable glasses (https://www.seeingwithsound.com/android-glasses.htm). Some researches from cognitive science also tried to explain how the brain processes the sound of VOICE [22,23]. The main drawback of VOICE is that it conveys color images to VIP, which is a very low level sonification, and they usually need a long time to learn how to infer 3D environments from the sound. Moreover, its information transfer rate is low (it needs one second to convert an image to sound). When environments change rapidly, the user may feel confused. In 2018, S. Spagnol et al. proposed a pathway sonification method using bubbles sounds [24]. They represented the parameter of depth images (such as the average depth value) with the parameter of bubble sounds (such as the depth of the bubble submerged in the liquid). A comparative wayfinding experiment was conducted in an indoor testing area and the results showed that their sonification method is more usable than VOICE in terms of obstacle avoidance and navigation. The research by S. Mascetti et al. focused on guiding VIP towards and over a pedestrian crosswalk [25]. They proposed three sonification methods (speech mode, stereo mode as well as mono mode) and performed both qualitative and quantitative evaluations to compare the effectiveness of these methods. However, their sonification methods were designed for crosswalk and cannot be used in general navigation.

Although there have been many works in the field of computer science and cognitive science to study the interaction methods for VIP, most of them did not analyze the effect of different types of scene detection algorithms and the corresponding sonification methods on the performance of ETAs. Based on this notion, we design three auditory-based interaction methods to convey different types of scene information to VIP. These three interaction methods can run in real time independently and are fully evaluated by twelve visually impaired participants in the user study. We find that as the degree of image processing deepens, the higher the level of information extracted from images, the smaller the cognitive burdens will be imposed on the brain of VIP, but at the same time, the lost environment details caused by image processing make it difficult for VIP to reconstruct the scene in their mind.

## 3. Sonification Method

In this section, we first introduce some preliminary of sonification and then present three scene sonification methods in detail. The first method is image sonification and it conveys raw depth images to VIP. The latter two methods, obstacle sonification and path sonification, utilize scene detection algorithms and convey the detection results to VIP.

### 3.1. Preliminary of Parameter Mapping Sonification

Parameter mapping sonification, a subcategory of sonification, focuses on mapping data parameters to sound parameters, and it is the most widely used sonification technique [26]. Auditory-based interaction for ETAs can be regarded as a specific application of parameter mapping sonification. Generally, parameter mapping sonification contains three basic parts: data parameters, sound parameters and the mapping function between these parameters. In this subsection, we present the sound dimension and the notation of mapping function briefly.

#### 3.1.1. Sound Dimension

Sound is generated by object vibration and propagates as a longitudinal wave. As a kind of wave, pitch and loudness (usually known as frequency and amplitude in physics) are the most basic sound parameters. Pitch is the most frequently used parameter in sonification because it has large dynamic ranges and high resolution [27]. Furthermore, pitch is also able to convey absolute values to VIP with some degree of musical training. Loudness is not as useful as pitch because generally people can only distinguish which one of the two sounds is louder but cannot determine the absolute loudness of a sound. Loudness also has lower resolution compared with pitch and is easily affected by environmental noise. For these reasons, loudness is usually used to convey two or three discrete levels or the changing trend of data. Then we consider temporal and spectral characteristics of sound. Timbre is a useful sound dimension and people can use timbre to distinguish what kind of musical instrument is playing if the pitch and loudness are the same. It is efficient to use timbre to represent different types of data. Tempo is the speed of a sound. A sound with fast tempo usually represents urgency, such as dangerous obstacles. In addition, people can also perceive the spatial location of a sound source through binaural hearing, especially for people with visual impairments [28]. It is natural to use spatial location to represent the spatial parameter of objects.

**Figure 2:**
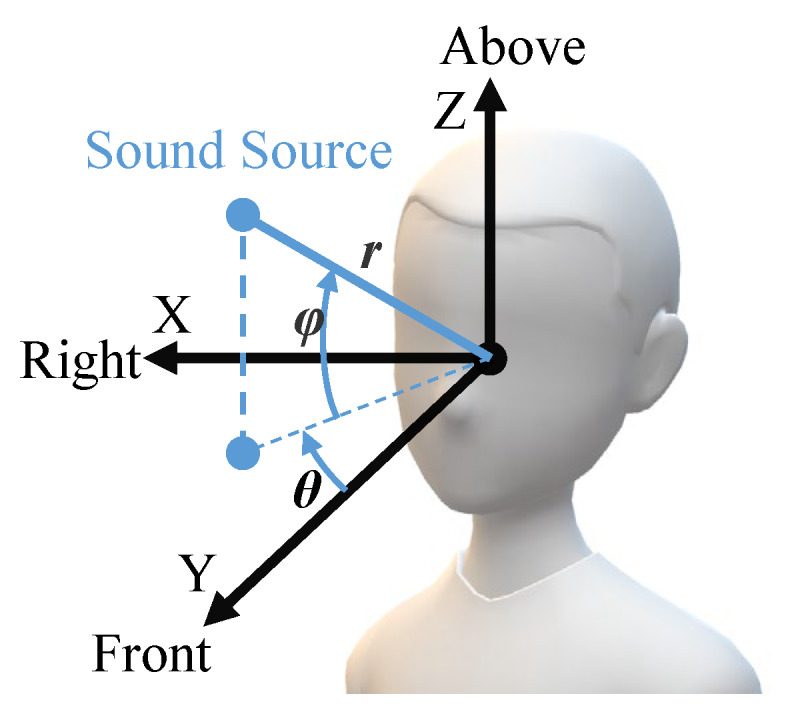
Right-handed spherical coordinate that used to represent the spatial location of sound sources.

Next, we introduce the unit of the above sound parameters. In order to take advantage of VIP’s prior knowledge of music, we use semitone (st), the smallest pitch interval in western tonal musical theory, instead of hertz (Hz) to measure pitch. For the sound used in our work, we declare the original pitch first and use the relative change measured in st to indicate pitch. Positive (negative) semitone indicates that the pitch increases (decreases) from the original pitch. Loudness is the subjective perception of sound pressure, and we use the gain or attenuation values measured in dB to quantify loudness. As for tempo, it is typically measured in Beats Per Minute (BPM). To represent tempo more intuitively, we use the time interval between two repeated playbacks of a sound to measure tempo. About spatial location, a right-handed spherical coordinate (r,θ,ϕ) is used to present location of sound source, where r≥0, $-180^{\circ }<\theta \leq 180^{\circ }$, and $-90^{\circ }<\phi \leq 90^{\circ }$, as shown in Figure 2. We spatialize the sound by adjusting the intensity of sound channels (stereo panning) [29]. People usually have high horizontal resolution in the front, thus θ ranging from $-90^{\circ }$ to $90^{\circ }$ is the most commonly used parameter of spatial location.

#### 3.1.2. Mapping Function Notation

We use piecewise linear functions to express mapping functions. Compared with other functions (e.g., sigmoidal function), piecewise linear function is more intuitive when adjusting thresholds. To avoid complex math descriptions, discrete pairs of points in braces $\left\{\left(d_{1},s_{1}\right),\ldots ,\left(d_{n},s_{n}\right)\right\}$ are used to represent the inflection point in a linear piecewise function, where $s_{i}\left(1\leq i\leq n\right)$ is the value in the sound parameter domain corresponding to $d_{i}\left(1\leq i\leq n\right)$ in the data parameter domain. If the data parameter or sound parameter is not a continuous quantity (such as timbre), we use square brackets $\left\{\left[d_{1},s_{1}\right],\ldots ,\left[d_{n},s_{n}\right]\right\}$ to represent the discrete mapping function, where $d_{i}$ and $s_{i}$(1≤i≤n) represent the value of the data parameter and sound parameter respectively.

### 3.2. Image Sonification

Distance information in depth images is crucial to obstacle avoidance. Thus, the basic idea is to convert depth images to sound directly. In this method, we regard each pixel in depth images as a sound source and design a rule that maps pixel parameters to sound parameters.

**Figure 3:**
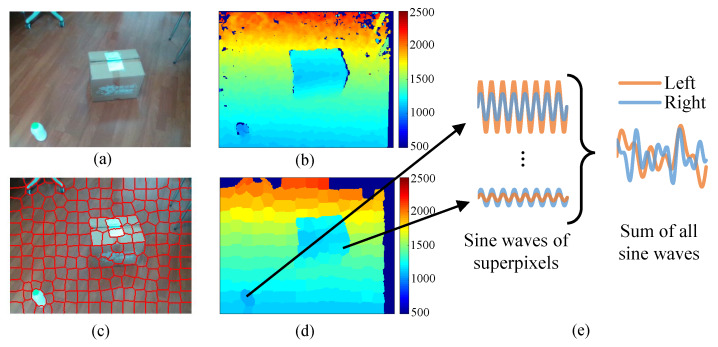
Image sonification. (**a**) Raw color image. (**b**) Raw depth image, where invalid depth values are indicated by dark blue. (**c**) The result of superpixel segmentation on color image. (**d**) Downsampled depth image guided by superpixels in (**c**). (**e**) Schematic of image sonification.

Raw depth images output by RGB-D cameras typically have thousands of pixels and rendering so many sound sources requires a lot of computing resources. In order to reduce the size of depth images without affecting small obstacles in depth images, a superpixel-based downsampling approach is employed. As shown in Figure 3, we first capture both depth images and color images with the same Field of View (FoV). Then the color image is segmented to *n* superpixels based on the Simple Linear Iterative Clustering (SLIC) superpixel algorithm [10]. SLIC evenly selects *n* pixels in the image as initial clustering centers, and iteratively performs k-means clustering algorithm to group pixels with similar color into a superpixel. The resolution of our color images is 320×240. We set n=192(16×12) and thus on average every 20×20 pixels are represented by one superpixel. Finally, the segmentation results are applied to the depth image and the depth value of a superpixel is calculated by the mean depth of all pixels in it. Through this manner, the number of pixels, i.e., the number of sound sources, is reduced greatly and the depth information of small obstacles is also kept. Note that if most pixels in a superpixel have no depth value, the superpixel will be labeled as invalid and do not produce sound.

Next, we introduce the sonification rule. Each superpixel has three parameters, the 2D centroid (x,y) in the pixel coordinate system and the depth value *D*. A sine wave with a specific pitch, loudness and 3D direction is used to represent a superpixel. The original pitch of sine waves is set to $A_{5}$ (880 Hz). Assuming that the maximum amplitude that the hardware can play is 1, the original amplitude of sine waves is set to 1/n to ensure that the sum of all sounds does not exceed the maximum. The mapping functions from the parameters of superpixel to the parameters of sound are given in Table 1 based on the units described in Section 3.1.1.

The horizontal FoV of the RGB-D camera we used is about $60^{\circ }$, which is less than the azimuth range ($-90^{\circ }$ to $+90^{\circ }$) of virtual 3D sounds. It means that the azimuth of virtual 3D sound sources is not strictly aligned with the objects in the FOV. We believe this misalignment is acceptable because VIP primarily determine the position of obstacles by turning the head and listening to the change of sound in the azimuth dimension, and a larger azimuth range of virtual 3D sounds makes this easier. Another potential issue in the azimuth dimension lies in the sounds of two separate obstacles from the left and right may be combined as a sound at the center. In practical, VIP usually further check the distribution of obstacles by turning the head slightly, thus this issue may not have much impact on the environmental perception. The inflection point of depth-to-loudness mapping function is (1.5 m, −50 dB). It means the loudness with a depth value greater than 1.5 m is very low, and if the depth value is less than 1.5 m, the loudness will increase significantly, which suppresses the sound of the ground and highlights small obstacles on the ground. In terms of the mapping from vertical coordinates to pitch, we set the superpixel at the lower position to a higher pitch. The main reason of this choice is that the bottom of the image usually contains the ground, which is more crucial for obstacle avoidance. Compared with low-pitch sounds, high-pitch sounds can better remind VIP to pay attention to the obstacles on the ground.

Using this sonification method, VIP may not discriminate each superpixel clearly but they are able to perceive the entire image simultaneously. Figure 3e shows an example of image sonification. There is a small bottle on the left and a box on the right, VIP will hear two main sounds mixed together, one at higher pitch and loudness on the left and the other at lower loudness and pitch on the right.

### 3.3. Obstacle Sonification

Obstacle sonification conveys obstacle information extracted from images to VIP and contains less redundant information compared with the sonification of raw depth image. We have done some research on detecting the ground and obstacles using RGB-D cameras for VIP and proposed a simple sonification method utilizing the sound of multiple musical instruments to represent the detected ground in our previous work [11]. In this work, we reorganize some key techniques to improve the efficiency of the obstacle detection algorithm and focus on the design of sonification methods.

We only rely on depth images and utilize point cloud-based techniques to detect the ground and obstacles. First, we calculate the point cloud from the depth image and adjust it to the local east-north-up (ENU) coordinate system, formulated as:

(1) $$\left[\begin{array}{c} X\\ Y\\ Z \end{array}\right]=R{\left[\begin{array}{ccc} f & 0 & c_{x}\\ 0 & f & c_{y}\\ 0 & 0 & 1 \end{array}\right]}^{-1}\left[\begin{array}{c} x\\ y\\ 1 \end{array}\right]d$$

where *d* is the depth value at (x,y) in the depth image, $f,(c_{x},c_{y})$ are the focal length and principle point of depth camera respectively, R is the rotation matrix of camera acquired from the attitude sensor, and ${\left[\begin{array}{ccc} X & Y & Z \end{array}\right]}^{T}$ represents the 3D point in the ENU coordinate system.

**Figure 4:**
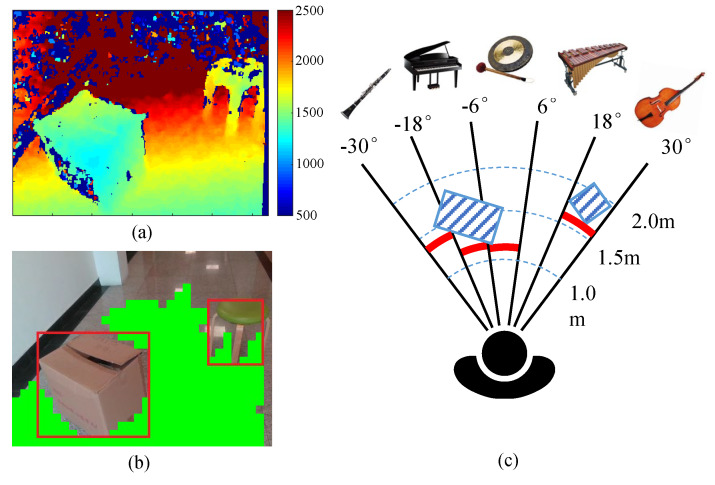
Obstacle sonification. (**a**) Raw depth image. (**b**) The results of ground and obstacle detection, where green masks represent the ground, and the detected obstacles are highlighted by red bounding boxes. (**c**) Sonification scheme, where obstacles are indicated by blue striped blocks and the obstacle distance of each section is indicated by red bold lines.

Then we use a two-stage method to detect the ground and obstacles. At the first stage, a RANdom SAmple Consensus (RANSAC) algorithm [30] is utilized to coarsely detect the largest plane in the point cloud. We randomly select three points from the point cloud and calculate a plane equation in 3D space. The remaining points are used to evaluate this plane equation. If the distance of a point to this plane is less than a threshold, the point will be regarded as inlier. After *m* iterations (we set m=20), the plane with most inliers is determined as the candidate of ground plane and all inliers are labeled as the ground. At the second stage, we further refine the ground points by checking if their normal vector is perpendicular to the ground. We divide depth images into 10×10 patches evenly. If most pixels in a patch are labeled as ground, its normal vector will be estimated through a least square method. Assuming there are *m* points in the patch and the coordinate of the *i*th point is $X_{i}$, $Y_{i}$ and $Z_{i}$, the 3D plane parameters A,B,C,D can be calculated by solving the equation in Equation (2) and the normal vector V is given in Equation (3). If the normal vector of a patch has a low component in the vertical direction, it will be discarded. The remaining patches are the detected ground, as shown in the green region in Figure 4b.

(2) $$\begin{array}{c} \left[\begin{array}{cccc} X_{1} & Y_{1} & Z_{1} & 1\\ \vdots & \vdots & \vdots & \vdots \\ X_{m} & Y_{m} & Z_{m} & 1 \end{array}\right]\left[\begin{array}{c} A\\ B\\ C\\ D \end{array}\right]=0 \end{array}$$

(3) $$\begin{array}{c} V=normalized({\left[\begin{array}{ccc} A & B & C \end{array}\right]}^{T}) \end{array}$$

Obstacle detection will be performed after the ground is successfully detected. We randomly select some pixels as seeds in the non-ground region of depth images and perform the seed region growing algorithm to expand the surrounding pixels with consistent depth value [31]. If the size of expanded region is larger than 10 cm, it is considered as a significant obstacle and its bounding box will be calculated to represent the obstacle. This simple and effective method can detect obstacles from environments quickly and robustly when the ground is detected. Some state-of-the-art algorithms, such as 3D dense surface mapping [7] and deep learning-based object detection [32], can get similar results but usually require more computing resources. Figure 4a,b show the raw depth image and the results of ground and obstacle detection projected on the color image, respectively.

Next we present the obstacles sonification rule that convert the detected obstacles to sound. We project the detected obstacles to a polar coordinate system on the ground and split the polar coordinate space into five sections in azimuth dimension, as shown in Figure 4c. A unique musical instrument is assigned to each section and we map the obstacle distance of each section to the parameters of musical instrument. The original pitch of these musical instruments is $A_{4}$ (440 Hz) and the detailed mapping rule is given in Table 2.

To make VIP clearly distinguish obstacles in different direction, we choose five different types of musical instruments to represent five sections. The instrument in the middle section is the gong, which has the most distinctive timbre and is quite easy to distinguish from the other instruments. The sound of the gong means there is an obstacle in the front and VIP cannot go straight. The musical instruments beside the gong is the piano and marimba. Their timbres are similar but not as prominent as the gong. The sound of the piano and marimba means there are obstacles on the left or right side of the front area and if VIP don’t adjust their walking direction according to the sound, there is still a risk of collision. The leftmost and rightmost musical instruments are clarinet and bass. They are string instrument and woodwind instrument respectively and is suitable for playing continuously. The obstacle represented by clarinet and bass will not block VIP from walking forward. In actual use, VIP should first distinguish different sections through the timbre and azimuth of musical instruments, and then estimate the distance of obstacles by loudness, pitch as well as tempo.

### 3.4. Path Sonification

Traversable area is the highest-level information compared with obstacle information and raw depth images since VIP can directly know which direction is safe to walk based on this information. In [33], we developed a polarized RGB-D framework to detect water hazard regions on the ground and proposed the concept of traversable distance curve to lead VIP to find the pathway in outdoor environments. In this work, we redesign the sonification method that convert the traversable distance curve to sound.

**Figure 5:**
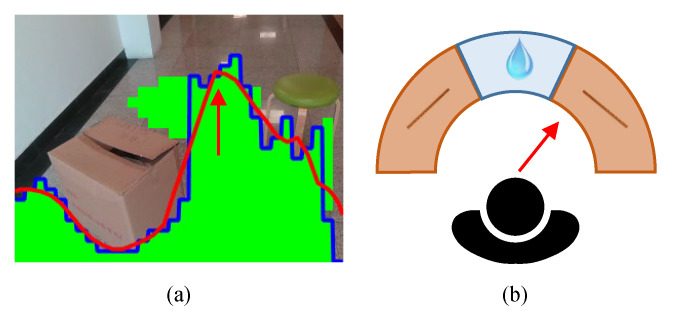
Path sonification. (**a**) Traversable distance curves, where green masks represent detected ground, and the blue curve and red curve represent respectively the raw traversable distance curve and the smoothed curve. The red arrow denotes the most-traversable direction. (**b**) According to the most-traversable direction, either flute sound or water drop sound will be played.

Traversable distance curve is generated from the detected ground described in Section 3.3. It is defined as the distance the ground extends in every direction. We smooth this curve through a 7-point sliding-average filter and define the most-traversable direction as the direction with highest value in the curve, as shown in Figure 5a. The most-traversable direction usually changes continuously as VIP turn their head. Therefore, we use flute, an instrument that suitable for playing continuously, to indicate the most-traversable direction. The original pitch of the flute sound source is $A_{4}$ (440 Hz). Additionally, if the pathway is in the front, a water drop sound is used to feedback the traversable distance in the front. The detailed parameter mapping rule is given in Table 3.

The azimuth of flute indicates the direction that VIP need to turn to, and the more VIP deviate from the most-traversable direction, the louder the flute plays and the pitch also becomes higher. Through this manner, VIP can acquire the most-traversable direction accurately. The sound of water drop represents the traversable distance in the front and it is only played when the deviation between VIP’s orientation and the most-traversable direction is small enough (less than 10 degrees). In other words, the sound of water drop is a sign of safety. VIP can also estimate how far they can safely walk ahead through the tempo of water drop.

## 4. Experiment

In order to validate the usability of three proposed sonification solutions, a comprehensive field experiment was performed with the attendance of VIP. Visually impaired participants were requested to walk through an area with some obstacles by three sonification methods and white cane respectively. Both qualitative results (subjects’ evaluation towards navigation experience) and quantitative statistics (the number of collisions, etc.) were collected to analyze the performance of sonification methods. In this section, we first introduce the implementation of our hardware system, and then explain the experiment process in detail.

### 4.1. System Implementation

**Figure 6:**
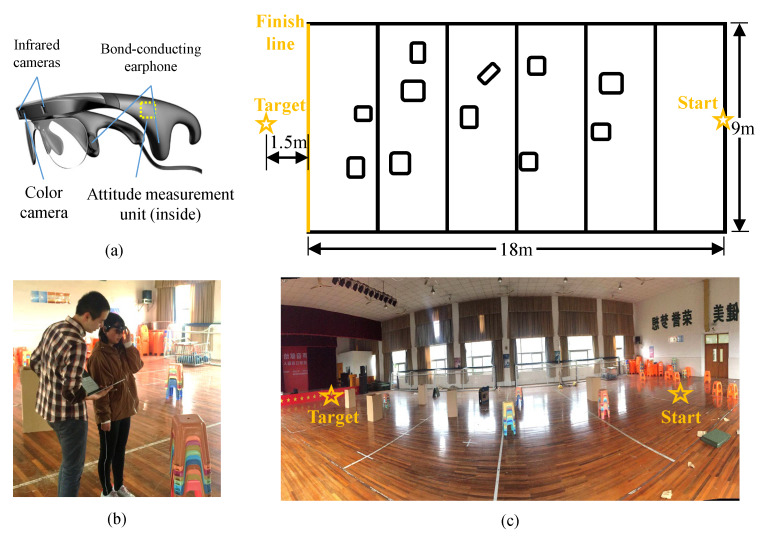
Field experiments. (**a**) Hardware implementation. A pair of smart glasses with an RGB-D camera and a bone conduction headphone. (**b**) A participant is training in the field with the help of us. (**c**) Diagram and panorama image of the experiment field, where obstacles are represented by little squares.

The wearable ETA used in the experiment is shown in Figure 6a. An RGB-D camera, an attitude sensor and a bone conduction headphone are integrated into a pair of smart glasses. The RGB-D camera is a commercial RGB-D camera of RealSense LR200 [34]. Its horizontal and vertical FoV are 59 degrees and 42 degrees respectively, which is adequate to assist VIP in most scenarios. The attitude sensor is a standalone system that utilize inertial and magnetic sensors to estimate 3D orientation in the ENU coordinate system [35]. We use bone conduction headphones since they do not prevent VIP from perceiving sound from environments. The glasses are connected to a laptop with a CPU of Intel Core i5-7200U @2.5 GHz and 8 GB RAM through an USB 3.0 cable.

Next we introduce the software implementation. All musical instrument sound sources are from the Musical Instrument Samples Database [36]. FMOD (https://www.fmod.com), a sound effects engine, is used to play sound sources and change sound parameters. The frame rates of three sonification methods are 13 Hz, 8 Hz and 10 Hz, which can provide sufficient prompts when VIP walking.

### 4.2. Participants

Twelve visually impaired volunteers from a blind high school were invited to participate in the experiment. All participants have received complete primary education at school and have learned how to use a white cane. The basic information of each participant is listed in Table 4. Note that some of them were below 18 years old and we obtained the consent of their parents.

### 4.3. Training

Before the field experiment, we need to ensure that all participants have fully understood the sonification rules, so a training session was conducted first. We demonstrated the purpose and rules of each sonification method to participants, and then helped them put on the smart glasses and walked freely in the field, as shown in Figure 6b. By monitoring the captured image and the running status of the sonification program, we can tell them the meaning of the sound they are listening and how to utilize the sound to find a pathway. Furthermore, we also provided a concise guide to help them master three sonification methods as soon as possible, as shown below.

- Image sonification: this sonification method provides rich scene information. You need to determine the location and size of obstacles first, and then bypass them. Big obstacles, such as a wall, usually occupy most of the image and the sound usually contains multiple frequencies, while the sound of small obstacles, such as a bottle, is generally thin and crisp. You can confirm the size and position of obstacles by slowly turning your head.
- Obstacle sonification: this sonification method converts obstacle information to sound, thus mute means safety. The sound of the gong, piano and marimba indicates that there are obstacles in front of you and you should stop immediately and try another walking direction.
- Path sonification: this sonification method converts pathway information to sound. You should turn following the sound of flute, and go straight when hearing the water drop sound.

By touching obstacles while listening to the sound, participants were able to understand and adapt to the sonification rules. They could keep training until they had fully understood the sonification rules and passed a very basic test with a single obstacle. The time of learning and adaptation was recorded and used to measure the complexity of sonification methods.

### 4.4. Field Experiment

The field experiment was conducted in a goalball field. The size of the field was 18 m by 9 m. Some cartons and plastic stack stools were randomly placed in the field as obstacles, as shown in Figure 6c. Participants were requested to traverse the experiment field from the start point to the target point as quickly as possible by using three sonification methods and white cane respectively. A speaker that constantly playing music was placed at the target to lead subjects walking towards the right direction. For each method (three sonification methods and white cane), participants needed to finish the experiment three times, i.e., every participant needed to complete twelve experiments in total. In order to prevent participants from remembering the distribution of obstacles in the field, we moved the obstacles randomly before each trial. The order of using three sonification methods was also random to make the comparison fair. The time that participants walked from the start point to the finish line, the number of collisions as well as the number of deviating from the target (cross the side lines of the field) were recorded for further analysis.

### 4.5. Questionnaire Survey

After the experiment, a questionnaire survey was conducted to evaluate three sonification methods. The questionnaire consisted of five items rated on 7-point Likert scales ranging from 1 (strongly disagree) to 7 (strongly agree) for each sonification method:

- Item 1: (Scene representation) I can understand the size and position of obstacles through this sonification method.
- Item 2: (Navigation) I know if there are obstacles ahead and how to avoid them through this sonification method.
- Item 3: (Complexity) I feel relaxed and don’t have to concentrate all my attention on the subtle changes of sound.
- Item 4: (Comfort) I think the sound effects are not noisy and the device can be used for a long time.
- Item 5: (Overall) I like this sonification method.

Moreover, we also collected the feeling and suggestions for each sonification method as well as the most favorite method among three sonification methods and white cane method.

## 5. Results and Discussion

**Figure 7:**
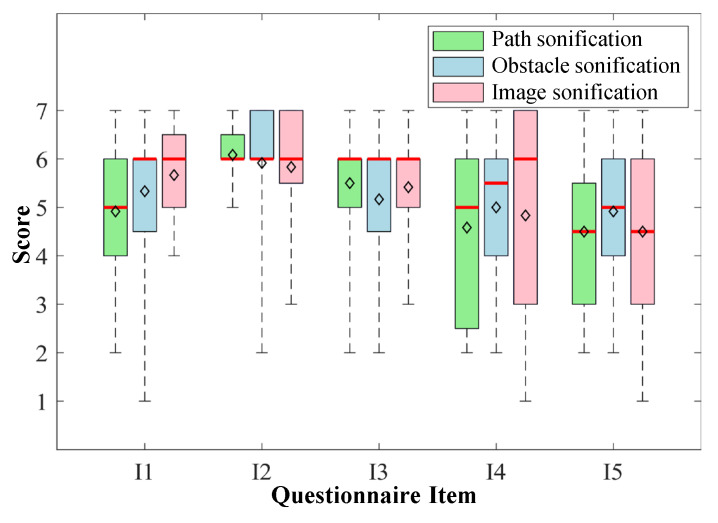
Box chart of the questionnaire results. I1 to I5 indicate five items of questionnaire for evaluating scene representation, navigation, complexity, comfort and overall satisfaction, and the score is based on the 7-point Likert scales, where 1 means strongly disagree and 7 means strongly agree. The median and mean values of each item are shown in red lines and diamond markers respectively.

The results of experiment and questionnaire are shown in Table 5 and Figure 7. In the following subsections, we compare the performance of three sonification methods and then analyze their characteristic in different aspects.

### 5.1. Sonification Versus White Cane

Mean completion time and number of failures in the field experiment can be used to evaluate the performance of a method. As shown in Table 5, the mean completion times of three sonification methods are 41.9 s, 42.5 s as well as 41.2 s, which are very close to each other. As a comparison, the mean completion time of using a white cane is 37.1 s, and it is a little shorter (about 5 s) than using sonification. A inferential statistics is conducted to further analyze this difference. Since participants have systematically learned how to use a white cane at school but only trained our methods for a few minutes, we perform an unequal variance t-test to identify if the completion time of white cane method is statistically shorter than the time of three sonification methods. The p values are 0.167, 0.136 as well as 0.192 respectively. This results show that the difference of completion time is not statistically significant, which means the walking speed of sonification method is not significantly lower than using a white cane.

Next, we consider the number of failures in the experiment. When using sonification methods, 14 failures occurred in all 36 trials, especially in the trials of the image sonification and obstacle sonification, while no failure occurred for white cane method. The result shows that using a white cane seems more safe. We think that the total number of failures when using sonification is also acceptable, and in addition five of the twelve participants did not make any mistakes in the experiment.

Although white cane performs better in the experiment, the three sonification methods also show their capability of conveying scene information and assisting VIP in this experiment.

### 5.2. Navigation Versus Scene Representation

All three sonification methods can provide enough prompts to assisting VIP, but the way VIP extract environmental information from sound and then decide which direction to walk is quite different. When using the image sonification and obstacle sonification, participants first understand the scene in the front and then infer which direction they should go. In contrast, when using path sonification, participants can directly know where is traversable and how far they can go, but the obstacle information is still unknown. This difference implies that path sonification is more suitable for navigation while the other two sonification methods are more inclined to help VIP understand environments.

The first two items of the questionnaire are designed to verify the above viewpoint. As shown in Figure 7, the I1 (Scene representation) score of image sonification is the highest, and the score of path sonification is the lowest, which support our viewpoint. As for the I2 in the questionnaire, there is also a trend that path sonification has the highest score, but the difference between them is very small.

We believe both of these two aspects are helpful for VIP. For example, if VIP don’t have a specific target and want to explore the surroundings, the ability of scene representation is more important obviously. But if VIP know the destination, the ability of navigation will be more helpful.

### 5.3. Complexity

The time of learning and adaptation in the training session can be used to measure the complexity of a sonification method. As shown in Table 5, the mean training time of three sonification methods are 5.8 min, 11.3 min as well as 14.5 min respectively. This result is as expected given the length of sonification guides described in Section 4.3. The training time of image sonification is very long, because this sonification method is the most abstract one and participants need more time to adapt to the sound of some typical scene. In contrast, path sonification is the simplest method and participants only need to remember one sentence: turn follow the flute sound and go straight when hearing the water drop sound.

The third item of the questionnaire is designed for evaluating this feature, but the result seems not to fully support this idea: the mean score of obstacle sonification is the lowest and the other two methods have similar scores. The main reason maybe the obstacles in the field are too simple, i.e., the shape of all obstacles is approximately cubic and the total number of obstacles is small, so participants only need to determine if there is an obstacle ahead and do not have to check the surrounding area carefully.

### 5.4. User Preference

The fourth and the fifth items of the questionnaire are used to investigate the subjective feelings of sound effects and the overall satisfaction of sonification methods. The variances of these two items are very large, especially for image sonification, as shown in Figure 7. The last column of Table 5 also shows the most favorite method among sonification methods and white cane method. The choices of participants are almost evenly distributed across four methods and not concentrate on a specific method (S1:S2:S3:W=2:3:3:3). In the post-experiment interview, we collected some feedback that why participants prefer one method to the others. Participant 8 said she was a music fan and sensitive to musical instruments. She liked the last two sonification methods because they utilized more musical instruments to represent pathways and obstacles. Participant 12 often played role-playing audio games, so he was very familiar with representing things through 3D sound. He performed well in the experiment and he liked image sonification method best because it can convey much more information than the other two methods. The discussion above indicates that there is no sonification method that is best suited for every VIP, and they need a period of adaptation to find the most suitable interaction method.

### 5.5. Navigation Failures

In terms of the number of failures, three sonification methods perform differently. All three failures of lost direction occurred when using path sonification (participant 1 and 9). The main reason is that path sonification regards the widest pathway as the preferred pathway, while the widest pathway is not always the correct direction to the target, which may cause participants to deviate from the target. An indoor positioning and navigation system, such as a WiFi-based positioning system in [37], can solve this problem and make path sonification more practical. On the contrary, when using the other two sonification methods, detection algorithms do not force participants to turn left or right, and the failure of lost direction did not happen.

Next, we discuss the reason of collisions. Most of collisions were caused by the narrow vertical FoV of our camera. If participants do not maintain the optimal angle by keeping their head at a lower position, obstacles will not be captured and collisions may occur. This is also a common problem of head-mounted ETAs. Moreover, the failure of scene detection algorithms may also cause collisions. The ground of our experiment field is reflective, so the images will be overexposed at certain angles, and in that case, scene detection algorithms may output incorrect results and causes collisions.

## 6. Conclusions and Future Work

In this paper, we propose three different auditory-based interaction methods that convey different levels of scene information to sound for assisting VIP. A field experiment is also conducted to evaluate the usability and difference of three sonification methods. The results show that all three methods are effective in scene representation. Moreover, we find that high-level scene information, such as pathway information, is useful for navigation and easy to learn, but it fails to represent surrounding details. On the contrary, low-level scene information, such as depth image, contains enough scene details, but the amount of information is large and it takes more time to learn and adapt. These conclusions can be used to guide the design of ETA and migrate to other fields, such as the sound feedback for virtual reality and augmented reality applications. The instability of scene detection algorithms and the inefficiency of interaction methods are still the bottleneck that limit the performance of ETAs, and there is still a long way to go in the field of helping VIP travel safely and independently.

In the future, we aim to design more robust scene detection algorithms by incorporating real-time semantic segmentation [38]. In addition, we also want to try to convey multiple levels of information to maximize the brain potential.

## Figures and Tables

**Table 1:** Parameter mapping rules of image sonification. Parameters of superpixel are mapped to the parameters of sine waves. The origin of the image coordinate system is at the top left corner of raw depth images at the resolution of 320×240.

Data Parameter	Sound Parameter	Mapping Function
Superpixel vertical coordinate	Pitch	{(0, −6 st), (240, +6 st)}
Superpixel horizontal coordinate	Azimuth	$\left\{\left(0,-90^{\circ }\right),\left(320,+90^{\circ }\right)\right\}$
Depth value	Loudness	{(0.2 m, 5 dB), (1.5 m, −50 dB), (3.5 m, −70 dB)}

**Table 2:** Parameter mapping rules of obstacle sonification. Sections in the polar coordinate system is mapped to the parameters of instrument.

Data Parameter	Sound Parameter	Mapping Function
Section index	Timbre	{[1,Clarinet],[2,Piano],[3,Gong],[4,Marimba],[5,Bass]}
Section index	Azimuth	$\left\{\left[1,-24^{\circ }\right],\left[2,-12^{\circ }\right],\left[3,0^{\circ }\right],\left[4,12^{\circ }\right],\left[5,24^{\circ }\right]\right\}$
Section distance	Loudness	{(0.2 m, 0 dB), (1.5 m, −40 dB), (3.5 m, −70 dB)}
Section distance	Tempo	{(0.2 m, 0.1 s), (1.5 m, 1 s), (3.5 m, 2 s)}
Section distance	Pitch	{(0.2 m, +6 st), (3.5 m, −6 st)}

**Table 3:** Parameter mapping rules of path sonification. Pathway information is mapped to the parameters of flute and water drop sound.

Data Parameter	Sound Parameter	Mapping Function
Most-traversable direction	Flute azimuth	$\left\{\left(-30^{\circ },-90^{\circ }\right),\left(+30^{\circ },+90^{\circ }\right)\right\}$
Most-traversable direction	Flute pitch	$\{(-30^{\circ }$, +12 st), (0${}^{\circ }$, 0 st), (+30${}^{\circ }$, +12 st)}
Most-traversable direction	Flute loudness	$\{(-30^{\circ }$, 0 dB), ($-10^{\circ },-20$ dB), ($-5^{\circ },-70$ dB), $(+5^{\circ },-$70 dB), (+10${}^{\circ },-20$ dB), (+30${}^{\circ }$, 0 dB)}
Traversable distance ahead	Water drop loudness	$\{(-10^{\circ },-$70 dB), ($-5^{\circ }$, 0 dB), (+5${}^{\circ }$, 0 dB), (+10${}^{\circ }$, −70 dB)}
Traversable distance ahead	Water drop tempo	{(1.5 m, 0.5 s), (2 m, 1 s), (3.5 m, 2 s)}

**Table 4:** Basic information of volunteers.

Subject ID	Gender	Age	Vision
1	female	18	total blind
2	female	18	total blind
3	male	17	low vision
4	male	18	total blind
5	male	18	total blind
6	male	19	total blind
7	female	19	total blind
8	female	17	low vision
9	male	17	low vision
10	male	18	total blind
11	female	19	total blind
12	male	17	total blind

**Table 5:** Experiment results. S1, S2 and S3 represent image sonification, obstacle sonification and path sonification respectively, and W represents the white cane method.

Subject ID	Training Time (min)			Mean Completion Time (s)				Number of Failures (Collision/Lost direction)				Most Favorite Method
	S1	S2	S3	S1	S2	S3	W	S1	S2	S3	W	
1	5	11	15	48.0	55.3	32.7	37.0	0/2	0/0	0/0	0/0	S2
2	6	12	11	28.4	30.2	39.2	31.2	1/0	1/0	0/0	0/0	S3
3	6	9	12	34.1	36.4	36.2	42.3	0/0	0/0	0/0	0/0	S2
4	6	10	14	38.1	36.7	28.9	30.7	0/0	0/0	0/0	0/0	S3
5	5	11	13	67.9	66.2	62.6	52.2	0/0	0/0	1/0	0/0	S2
6	5	13	12	35.6	33.0	35.2	33.5	0/0	0/0	0/0	0/0	W
7	5	12	17	36.8	37.6	42.4	32.8	2/0	2/0	0/0	0/0	W
8	6	15	15	33.4	36.0	36.3	32.5	0/0	0/0	0/0	0/0	S1
9	6	11	18	56.8	47.1	57.5	47.7	0/1	0/0	1/0	0/0	S1
10	7	9	16	52.9	60.1	47.6	36.0	0/0	2/0	0/0	0/0	S3
11	5	11	17	38.2	36.2	41.2	24.9	0/0	0/0	0/0	0/0	W
12	7	12	14	33.2	35.1	34.0	31.6	1/0	0/0	0/0	0/0	S3
Sum	/	/	/	/	/	/	/	4/3	5/0	2/0	0/0	/
Mean	5.8	11.3	14.5	41.9	42.5	41.2	37.1	/	/	/	/	/
SD	0.8	1.7	2.2	11.3	11.3	9.7	8.2	/	/	/	/	/
